# *Alternaria alternata* uses two siderophore systems for iron acquisition

**DOI:** 10.1038/s41598-020-60468-7

**Published:** 2020-02-27

**Authors:** Benjamin Voß, Frank Kirschhöfer, Gerald Brenner-Weiß, Reinhard Fischer

**Affiliations:** 1 Karlsruhe Institute of Technology (KIT) - South Campus, Institute for Applied Biosciences, Dept. of Microbiology, Fritz-Haber-Weg 4, D-76131 Karlsruhe, Germany; 2 Karlsruhe Institute of Technology (KIT) - North Campus, Institute of Functional Interfaces, Bioengineering and Biosystems, Hermann-von-Helmholtz-Platz 1, D-76344 Eggenstein Leopoldshafen, Germany

**Keywords:** Fungal biology, Fungal genetics

## Abstract

Iron is one of the most abundant elements on earth and essential for life. However, Fe^3+^ ions are rather insoluble and microorganisms such as fungi may use siderophores as strong chelators for uptake. In addition, free cytoplasmic iron is rather toxic and intracellular siderophores are used to control the toxicity. Siderophores are also important for iron storage. We studied two siderophore systems in the plant necrotrophic fungus *Alternaria alternata* and show that the non-ribosomal peptide synthase, Nps2, is required for the biosynthesis of intracellular ferricrocin, whereas Nps6 is needed for the formation of extracellular coprogen and coprogen B. Whereas *nps2* was dispensable for growth on iron-depleted medium, *nps6* was essential under those conditions. *nps2* deletion caused an increase in spore formation and reduced pathogenicity on tomato. Our results suggest that *A. alternata* employs an external and an internal siderophore system to adapt to low iron conditions.

## Introduction

Iron is an essential element for almost all organisms because of its role in cell proliferation and key metabolic processes like nucleotide biosynthesis or in energy production as co-factor in electron transfer processes. In contrast to its high abundance on earth, its bio-availability is very limited. Iron occurs in two different oxidation forms, solubilized ferrous iron (Fe^2+^) which is rare but easily accessible, and ferric iron (Fe^3+^) which is insoluble or bound to natural iron chelators and not accessible for cells. Microorganisms therefore developed different highly regulated strategies to get access to environmental iron^1^. Tight regulation though is very much needed because free iron generates hydroxyl radicals via the Haber-Weiss/Fenton chemistry, which are very harmful for the cells^2^. To acquire ferric iron from the environment, microorganisms use reductive and non-reductive strategies^3–5^. The reductive iron-uptake (RIA) pathway uses membrane-bound metalloreductases which reduce ferric iron to ferrous iron to enable uptake into the cells^6,7^. During iron-limited conditions, most bacteria and fungi synthesize and secrete siderophores in addition. These secondary metabolites chelate ferric iron with high affinity and specificity^1,5^. After uptake of the ferric-siderophore complex, iron is released by reduction or hydrolysis depending on the siderophore type. Free iron is then bound by other intracellular siderophores for storage and distribution.

Based on their chemical composition siderophores are divided into three groups, catechols, carboxylates and hydroxamates. With a few exceptions, fungi produce hydroxamate siderophores^3^. These can be assigned to four major families, rhodoturalic acid, fusarinines, ferrichromes and coprogens. The ascomycete *Aspergillus nidulans* for instance, produces ferricrocine and triacetylfusarinine, both derived from ornithine^8^. If the biosynthesis of both was prevented by deletion of the L-ornithine N^5^-oxygenase gene, the strain almost did not grow in the absence of additional iron added to the medium^9^. If only ferricrocine was missing, because of deletion of the non-ribosomal peptide synthase gene, *sidC*, the strain grew as well as wild type, but sporulation was largely affected^10^. These results show that the fine-tuning of the iron content in hyphae may be important for developmental decisions. The absence of the siderophore ferricrocine also caused an upregulation of antioxidant systems^9^. Iron acquisition has also been studied in the human pathogen *Aspergillus fumigatus*. Here it was shown that ferricrocine has also an iron-transport function and is important for virulence^11–13^.

Iron uptake and metabolism has only been studied in *Alternaria alternata* to some extent. *A. alternata* is an important post-harvest contaminant of food and feed and causes large annual losses^14^. *A. alternata* produces many different secondary metabolites, with alternariol as the name-giving compound^15–18^. During plant colonization the acquisition of iron may be an important factor and thus the production of siderophores may be essential for viability. Indeed, analysis of a non-ribosomal peptide synthase (NRPS), Nps6, revealed its role in the production of coprogen and a role in pathogenicity on citrus leaves^19^. Here, we identified a second NRPS, Nps2, and show that it is required for the formation of intracellular ferricrocin. Nps2 is also implicated in the control of developmental processes and the ability of *A. alternata* to colonize tomatoes.

## Methods

### Strains, plasmids and culture conditions

*A. alternata* strains were grown on mCDB (4% glucose, 0.1% yeast extract 0.1% NaNO_3_, 0,.025% NH_4_Cl, 0.1% KH_2_PO_4_, 0.025% KCl, 0.025% NaCl, 0.05% MgSO_4_ (7xH_2_O), 0.001% FeSO_4_, 0.001% ZnSO_4_, 1.5% agar) at 28 °C (180 rpm in liquid culture)^15^. To deplete iron from the medium 250 µM bathophenanthrolindisulfonic acid (BPS) was added to the medium. For the CAS-assay CAS (60.5 mg; MP Biochemical, Solon, OH, USA) was dissolved in 50 ml H_2_O and mixed with 10 ml of iron solution (1 mM FeCl_3_ and 6 mM HCl). Hexadecyltrimethyl ammonium bromide (HDTMA) (72.9 mg dissolved in 40 ml H_2_O) was carefully added to the CAS–iron solution. The solution was autoclaved and mixed with 750 ml of sterile mCDB^19^.

Split agar plates were filled on each side with the appropriate medium. The described strains were point inoculated at the same distance from the border and grown at 28 °C.

All strains are listed in Table 1, oligonucleotides in Table 2 and plasmids in Table 3.

**Table 1:** *A. alternata s*trains used in this study.

Strain	Genotype	Reference
ATCC 66981	Wild type	Christopher B. Lawrence, Virginia Tech, Blacksburg, VA, USA
SMW24	Δ*pyrG*	^20^
SBV2	SBV2, ∆*nps2* Δ*pyrG* sMW24 after transformation with pBV2 and pBV3	This study
SBV3	SBV3, ∆*nps6* Δ*pyrG* sMW24 after transformation with pBV4 and pBV5	This study
SBV4	SBV2 SBV3, ∆*nps2* Δ*pyrG* ∆*nps6* SBV2 after transformation with pBV4 and pBV5	This study
SBV5	SBV2 after recomplementation with *pyr-4* from *N. crassa*	This study
SBV6	SBV3 after recomplementation with *pyr-4* from *N. crassa*	This study
SBV7	SMW24 after recomplementation with *pyr-4* from *N. crassa*	This study

**Table 2:** Oligonucleotides used in this study.

Oligonucleotide	Sequence 5′ → 3′
Crispr fw 2.0	GGTCATAGCTGTTTCCGCTGA
Crispr re 2.0	TGATTCTGCTGTCTCGGCTG
*nps2* Proto fw1	GTCCGTGAGGACGAAACGAGTAAGCTCGTCGAGC GACCTTCGATTCTGAGGT TTTAGAGCTAGAAATAGCAAGTTAAA
*nps2* 6 bp re1	GACGAGCTTACTCGTTTCGTCCTCACGGACTCATCA GGAGCGACGGTGATGTC TGCTCAAGCG
*nps2* Proto fw2	GAGTAAGCTCGTCTTTGCGTCCGTGAGGACGAAAC GTGGACTGTCACCTGTTTTAGAGCTAGAAATAGCAAGTTAAA
*nps2* 6 bp re2	GACGAGCTTACTCGTTTCGTCCTCACGGACTCATCA GTTTGCCCGGTGATGTCT GCTCAAGCG
*nps6* Proto fw1	GAGTAAGCTCGTCGATTGCCTGCTGTCCGCATTGTT TTAGAGCTAGAAATAGCAAGTTAAA
*nps6* 6 bp re1	GACGAGCTTACTCGTTTCGTCCTCACGGACTCATCAGGATTGCCGGT GATGTC TGCTCAAGCG
*nps6* Proto fw2	GTCCGTGAGGACGAAACGAGTAAGCTCGTCTACTG GCAAAACCTCCTCTCGTTTTAGAGCTAGAAATAGCAAGTTAAA
*nps6* 6 bp re2	GACGAGCTTACTCGTTTCGTCCTCACGGACTCATCA GTACTGGCGGTGATGTCTGCTCAAGCG
*nps2* fw 1	ACCCCACAGCAAGAGTCAGTCAGTCTTAATATGATTCAAGAAAACGACGCAAAC
*nps2* rev 1	AATGCAAAGTAAACTGGAGGCTTC
*nps2* fw 2	AGCTGAGAAGCCTCCAGTTTA
*nps2* rev 2	CATCCACTTGGCTTCATCTAG
*nps2* fw 3	CAGGTCCGGCTAGATGAAG
*nps2* rev 3	GCTACTACAGATCGACTGACTGACTTTAATTTACAACTCTTCGAGTTGTCTCC
*nps2-*test-fw	CCAATCACCGTGCTGCAGT
*nps2-*test-rev	GAGCTTTCTCCAGTGTAAGC
*nps6-*test-fw	CGAGGCTCGAGCAAAAATCC
*nps6-*test-rev	GGATACGTTGTCTCGAACTTC
*nps2-*qpcr-fw	CCACAGCAAGAGTCAGTCA
*nps2-*qpcr-rev	TGATCAAATGGCCGCACAAG
*nps6-*qpcr-fw	CGAGTTTGAGGTCACAGATCA
*nps6-*qpcr-rev	TCAAAAGTCCACTCGTGTGTC
*AaltH2B*-qpcr-fw	ACAAGAAGAAGCGCACCAAG
*AaltH2B*-qpcr-rev	CGTTGACGAAAGAGTTGAGAAT

**Table 3:** Plasmids used in this study.

Plasmid	Content	Reference
pFC332	*tef1*(p)::*cas9*::*tef1*(t); *gpdA*(p)::*gpdA*(t); *hph*; *ampR*; AMA1	Uffe H. Mortensen, Technical University of Denmark
pFC330	*tef1*(p)::*cas9*::*tef1*(t); *gpdA*(p)::*gpdA*(t); Af*pyrG*; *ampR*; AMA1	Uffe H. Mortensen, Technical University of Denmark
pFC334	*tef1*(p)::*cas9*::*tef1*(t); *gpdA*(p)::sgRNA-*AnyA*::*gpdA*(t); Af*pyr4*; *ampR*; AMA1	Uffe H. Mortensen, Technical University of Denmark
pBV2	*tef1(p)::cas9::tef1(t); gpdA(p):: AapNPS2- sgRNA cassette1::trpC(t); hph; ampR; AMA1*	This study
pBV3	*tef1(p)::cas9::tef1(t); gpdA(p):: AapNPS2- sgRNA cassette2::trpC(t); hph; ampR; AMA1*	This study
pBV4	*tef1(p)::cas9::tef1(t); gpdA(p):: AapNPS6- sgRNA cassette1::trpC(t); hph; ampR; AMA1*	This study
pBV5	*tef1(p)::cas9::tef1(t); gpdA(p):: AapNPS6-sgRNA cassette2::trpC(t); hph; ampR; AMA1*	This study
Efimov-gpdA(p)	*gpdA(p)::trpC(t); ampR; pyr-4*	^31^

### Spore quantification

After the strains were grown on their respective media for different time points, the spores were scraped off the colony surface with a spatula in a defined volume of sterile water. The spore suspension was then filtered and the spores counted in a Neubauer counting chamber. Every measurement at every time point of each strain was repeated at least three times.

### Protoplast transformation of *A. alternata*

Fungal spores were harvested from a mCDB culture plate and inoculated into 100 ml liquid mCDB for overnight cultivation at 28 °C and 180 rpm as described before^15^. The mycelium was harvested by filtering, washed with 0.7 M NaCl and digested in a Kitalase (Wako Chemicals) suspension (150 mg in 15 ml 0.7 M NaCl) for 1 h with soft shaking at 100 rpm at 30 °C. Protoplast quality and quantity were checked via microscopy. Protoplasts were separated from cell fragments by filtering through two layers of Miracloth and precipitated at 680 g for 10 min at room temperature. The Kitalase solution was discarded and the protoplasts were washed once with ice cold 0.7 M NaCl and the centrifugation step was repeated. Afterwards the protoplasts were resuspended in 100 µl STC (1 M sorbitol, 50 mM CaCl_2_, 50 mM Tris-HCl, pH 8). 5–10 µg of Plasmid DNA were added to the protoplasts followed by a 10 min incubation on ice. DNA uptake was induced with a heat shock at 42 °C for 5 min and, after a 5 min incubation step on ice, 800 µl 40% PEG (40% polyethylene glycol [PEG] 4000, 50 mM Tris-HCl [pH 8], 50 mM CaCl_2_) was added to the protoplasts, followed by 15 min incubation at room temperature. The suspension was mixed with 50 ml warm regeneration medium (342.7 g/l sucrose; 0.5 g/l casein hydrolysate; 0.5 g/l yeast extract; 7.5 g/l agar) and distributed to two petri dishes. After overnight incubation at 28 °C the transformation plates were overlayed with 15 ml warm regeneration medium containing hygromycin (80 µg/ml) and again incubated until colonies were formed.

### Deletion of siderophore biosynthetic genes

For the design of the deletion constructs two protospacer sequences per gene were chosen to produce two different sgRNAs from the respective constructs. The strategy is based on previous work^15,20^. The sequences were introduced into the sgRNA-constructs by PCR using pFC334 as a template to obtain two PCR-fragments per protospacer that were cloned into pFC332 (first protospacer) and pFC330 (second protospacer) respectively (Table 4). For the PCR the oligonucleotides crispr fw 2.0 and crispr re 2.0 were paired with the oligonucleotides that contained the gene specific sequences “gene-6bp re 1 or 2” and “gene-Proto fw 1 or 2” (Table 3).

**Table 4:** Display of the deletion events using the CRISPR/Cas9 strategy.

Strain	WT sequence	Mutant sequence	Mutation
SBV2 (Δ*nps2*)	…[273]**AGAACTATACATAGTCTTAT**[292]… [8594]**TTTGCCGTGGACTGTCACCT**[8613]… → *nps2*: 16273 bp, 5372 aa	…[260]TGCCTCAGTCTCC **AGAACTATACATAGTCT**------------**CCT**TGGAAGATACGTCAGA CTCA [8633]…	8321 bp deletion 2757 aa deletion
SBV3 (Δ*nps6*)	…[666]**GATTGCCTGCTGTCCGCATT**[685]… [5184]**TACTGGCAAAACCTCCTCTC**[5203]… → *nps6*: 5930 bp, 1960 aa	…[650]CCTATTTAGCGACCCT**GATTGCCTGCTGTCCGC**–… ---------------**CTC**CGGCTCCCAG ATGTCTATCA [5223]	4518 bp deletion 1506 aa deletion
SBV4 (Δ*nps2* Δ*nps6*)	*nps2:* …[273]**AGAACTATACATAGTCTTAT**[292]… [8594]**TTTGCCGTGGACTGTCACCT**[8613]… *nps6*:…[666]**GATTGCCTGCTGTCCGCATT**[685]… [5184]**TACTGGCAAAACCTCCTCTC**[5203]…	*nps2:* …[260]TGCCTCAGTCTCC**AGAACTATACATAGTCT**--------------- ----------**CCT**TGGAAGATACGTC AGACTCA [8633]… *nps6:* ….[650]CCTATTTAGCGACCCT **GATTGCCTGCTGTCCGC**--------------------**CTC**CGGCTCCCAGA TGTCTATCA[5223]……	8321 bp deletion 2757 aa deletion 4518 bp deletion 1506 aa deletion

The coordinates of the displayed sequences (protospacer in bold) are given in distances from the ATG. The deletion event is indicated in the scheme of the protein (Mutation) as hatched box.

### RNA isolation and quantitative real time PCR

Conidia were inoculated with a loop on the surface of 20~25 ml of complete liquid medium mCDB in a Petri dish as described^21^. Mycelia were harvested, frozen in liquid nitrogen and stored at −80 °C until RNA isolation. To isolate RNA, frozen mycelia were ground into powder, and total RNA isolated using the E. Z. N. A. Fungal RNA Mini Kit (VWR, Darmstadt, Germany). RNA was quantified and an aliquot treated with DNase I. RNA samples were diluted to a final concentration of 50 ng/μl. Quantitative real time PCR experiments were performed to determine relative mRNA abundance using SensiFAST SYBR & Rad. Each reaction of 25 μl contained 0.2 μl of RT enzyme, 0.2 μM of primers and 100 ng of total RNA. The cycle included 10 min at 50 °C for the reverse transcription reaction, followed by 5 min at 95 °C for its inactivation and 40 PCR cycles (10 s at 95 °C, and 1 min at 60 °C). After each PCR, we performed melting curve analyses to show the specific amplification of single DNA segments and the absence of non-specifically amplified DNA. The results for each gene were normalized to the corresponding results obtained with histone *H2B*. Oligonucleotides are listed in Table 2.

### Metabolite extraction

For the extraction of secondary metabolites *A. alternata* strains were grown for seven days in 100 ml liquid medium shaking at 180 rpm and 28 °C^15^. Mycelium was harvested by filtering, washed with sterile H_2_O and lyophilized overnight. The dried mycelium was extracted with 10 ml 1:1 water/acetonitrile for one hour at room temperature. The supernatant was evaporated using a SpeedVac centrifuge. The pellet was extracted with dichloromethane for one hour at room temperature and then again evaporated. The final pellet was resuspended with methanol. The liquid phase was evaporated using a SpeedVac and then stepwise extracted with dichloro-methane and evaporated again until the final resuspension was done with methanol.

### Mass spectrometry (ESI-Q-ToF)

Mass spectrometric analyses were done using an X500R™ high resolution ESI-Q-ToF mass spectrometer (Sciex, Toronto, Canada) equipped with an electrospray ionization (ESI) source operating in the positive ion mode as described before^15^. The ion source was heated up to 450 °C and an ionspray voltage of 5300 V was applied. MS-experiments were carried out at a collision energy (CE) of 10 V and a declustering potential (DP) of 80 V. The acquisition range was 100 to 1200 m/z. In all experiments nitrogen gas 5.0 was used as nebulizer, curtain and collision gas. The resulting chromatograms were evaluated with SCIEX OS 1.5 instrument software.

### Liquid chromatographic separation (HPLC)

The samples were separated by HPLC (Agilent 1100, Waldbronn, Germany) using a Hypersil ODS RP C-18, 3 μm, 4 (i.d.) × 100 mm column (Thermo Fisher Scientific, Karlsruhe, Germany)^15^. A total runtime of 30 min was applied, starting at 10% of eluent A (CH_3_CN with 0.1% HCOOH) and 90% of eluent B (H_2_O with 0.1% HCOOH) for 2 min, followed by a linear gradient to 90% of eluent A over 18 min, which was held for 4 min and switched back to the starting conditions to equilibrate the column for the next run. The flow rate was constant at 500 μl/min and all analyses were carried out using a sample volume of 20 μl.

### Infection assays

The strains were pre-grown for 7 days at 28 °C on 60 mm petri-dishes (Greiner Biolabs) containing mCDB medium^15^. For inoculation of tomatoes spores from 7-day old pre-cultures were suspended in Tween salt solution (1 g Tween 80/l, 9 g NaCl/l). Tomatoes were surface-disinfected with 70% ethanol and carved with a sterile scalpel (1 cm). Afterwards 10.000 spores of the respective suspension have been carefully applied into the wound using a sterile pipette tip. The tomatoes were then incubated for one week at 20 °C. All experiments were done in biological triplicates.

## Results and Discussion

### The non-ribosomal peptide synthases Nps2 and Nps6 are required for ferricrocin and coprogen siderophore formation

**Figure 1:**
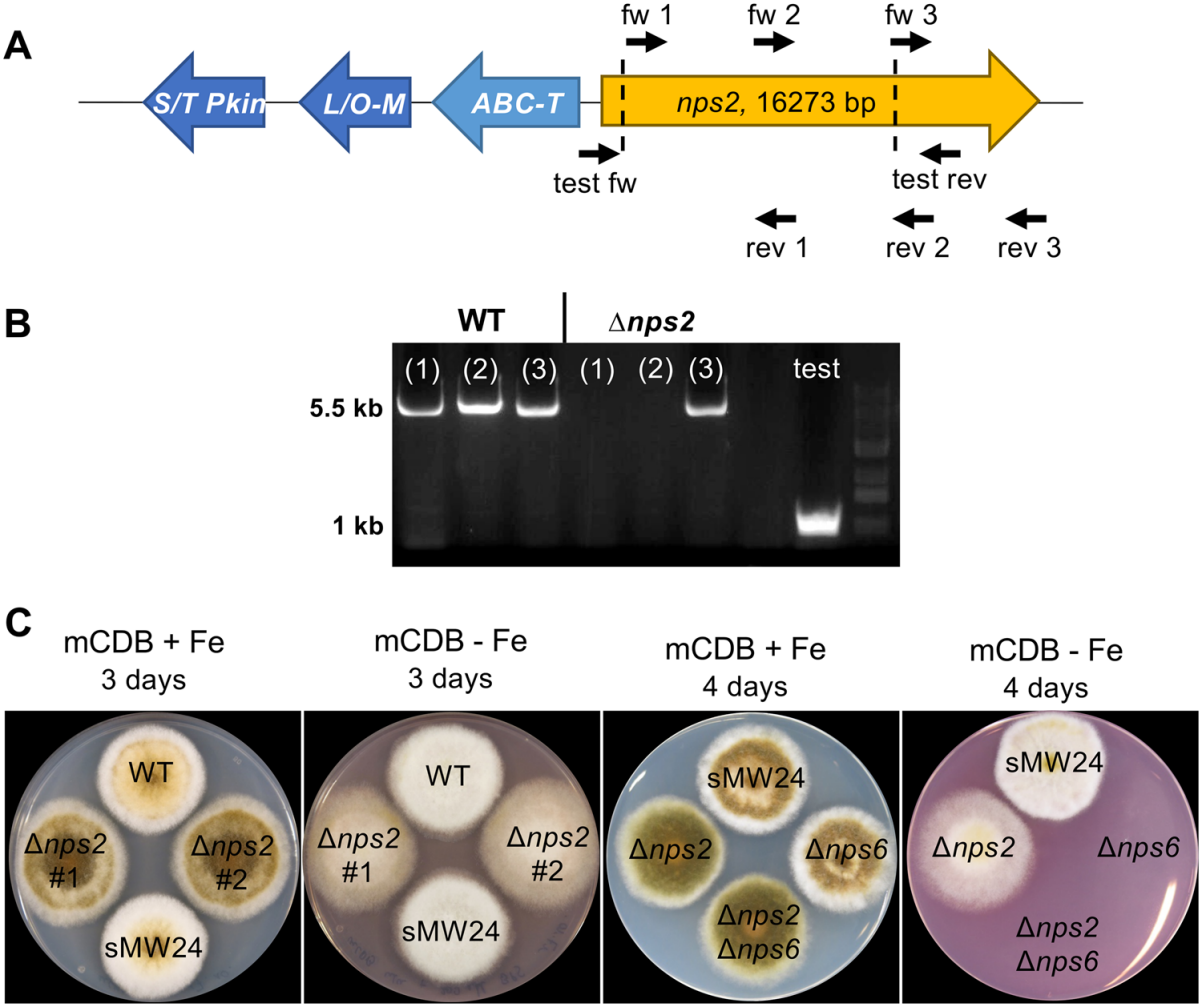
Deletion of *nps2* and *nps6* in *A. alternata*. (**A**) Analysis of the nps2 gene cluster with antiSMASH showed that three additional genes (ABC-Transporter = ABC-T, serine/threonine kinase = S/T Pkin, lysine/ornithine-monooxygenase = L/O-M) might play a role in siderophore biosynthesis. The oligonucleotides used to amplify pieces of the open reading frame are indicated above and below the scheme (nps2 fw1 with nps2 rev1 for fragment 1; nps2 fw2 with nps2 rev2 for fragment 2; nps2 fw3 with nps2 rev3 for fragment 3). Primers test fw and test rev were used to amplify the region with the deletion (test in (**B**)) (Table 2). The deleted region is indicated by dashed lines. (**B**) PCR analysis of genomic DNA from wild type (WT) and the *nps2-*deletion strain SBV2 (#1) using primers indicated in (**A**). The three fragments are indicated as (1), (2) and (3) in each lane. The PCR product amplified to show the deletion event is named as “test”. (**C**) Growth of WT (SMW24), *nps2* (SBV2, two independent deletion strains)*, nps6* (SBV3) and the *nps2/nps6*) SBV6) double deletion strain on CDB medium (plus uracil and uridine) plus and minus 250 µM BPS.

We identified five putative NRPS-encoding genes in the genome of *A. alternata* strain ATCC66981^22^, two of which, *nps2* and *nps6*, were studied in detail in this work. The open reading frame of *nps2* consists of 16,270 bp disrupted by three small introns at positions 664–713, 13,077–13,121 and 16,080–16,138 (confirmed by RNAseq data). The derived polypeptide consists of 5,372 amino acids (Fig. 1). NRPSpredictor2 revealed four adenylation domains with the first one (189 aa-337 aa) predicted to bind alanine (LSI score 0.447) or lysine (LSI score 0.371) or ornithine (LSI score 0.346) (https://bio.tools/NRPSpredictor2). Ornithine is essential for hydroxamate siderophore biosynthesis. Using antiSMASH, the genome region adjacent to *nps2* was analyzed for other genes putatively involved in siderophore biosynthesis (https://fungismash.secondarymetabolites.org/#!/start). We identified a gene encoding an ABC-transporter (possibly involved in secretion or transport of a siderophore into an organelle), a gene encoding a serine/threonine kinase, and a lysine/ornithine monooxygenase (a key enzyme in siderophore biosynthesis) (Fig. 1A). The similarity of the Nps2 protein with an overall of 27% identity to *A. nidulans* or *A. fumigatus* SidC, although quite low, further suggested that Nps2 is responsible for ferricrocin biosynthesis^9^.

The *nps6* gene of *A. alternata* had been described before as an intron-less open reading frame comprised of 5328 bps^19^. Nps6 is responsible for the biosynthesis of a N-dimethyl coprogen siderophore. However, the published data showed some discrepancies to our own results. We used RNAseq data for comparison with the genomic DNA sequence and identified one 47 bp intron at position 475 in the 5927 bp long open reading frame. The coding region of *nps6* is hence 5880 bp long and codes for a 1960 amino acids long protein (data not shown). The protein is thus 184 amino acids longer than the published version, although it has to be considered that a different *A. alternata* isolate had been used in the previous study^19^. It is also not clear if the authors analyzed only the DNA sequence or indeed showed that the open reading frame did not contain any intron. In any case the published open reading frame with 5328 bp is significantly shorter than the one described here. The nucleotide sequence of *nps2* and *nps6* reported here are available in the Third Party Annotation Section of the DDBJ/ENA/GenBank databases under the accession numbers MN939780 and MN939781, respectively.

In order to investigate the roles of the *nps2* and the *nps6* genes and the potential functional interplay of the produced siderophores, knock-out strains were generated, including a double-deletion strain. To this end we used the CRISPR/Cas9 system with two protospacer sequences as established recently^15,20^. In order to generate a *nps2* mutant strain, we used the *pyr4*-deletion strain SW24 and transformed it with the plasmids pFC330 and pFC332 (Fig. 1, Table 4). One plasmid contained a hygromycin resistance cassette, the other one the *pyrG* gene from *A. fumigatus*, and each one protospacer sequence (Fig. 1). 24 transformants were screened by PCR and three transformants showed an expected 1 kb band using primers outside the putative deletion event (Fig. 1B). In one of the three transformants further PCR experiments revealed that it was a heterokaryon. The remaining two putative *nps2-*deletion strains were used for sequence analysis. This proved the expected deletion of 8321 bp in strain SBV2 (no. 1 and 2) (Table 4). After the successful deletion, the strains were propagated on medium without hygromycin and in the presence of uracil and uridine. Because the CRISPR/Cas9 plasmids contain an AMA sequence for self-replication of the plasmids, the plasmids get lost in the absence of a selection pressure^23^. This was proven by growth tests on corresponding medium. The procedure guarantees that the Cas9 protein is not present constantly and cannot cause off-target effects. In order to produce an autotrophic *nps2*-deletion strain, SBV2 was transformed with the *pyr4* gene of *N. crassa*. This new strain, SBV5 was used in the virulence assays (see below). All other assays were performed with medium supplemented with uracil and uridine. Both *nps-2*-deletion strains, SBV2 and SBV5, did not have any vegetative growth defect in comparison to the wild type independent of the iron content of the medium.

In a similar way, a *nps6*-deletion strain and a *nps2-nps6* double-deletion strain were produced. The two plasmids pBV4 and pBV5 each containing one *nps6-*specific protospacer were transformed in the Δ*pyr4* strain SMW24 and in the Δ*nps2* strain SBV2. From each transformation 24 transformants were tested. The analytical PCR of SMW24 transformants revealed two which showed the promising 1 kb band. One was again heterokaryotic and was discarded. Hence one *nps6*-deletion strain (SBV3) with 4518 pb lacking, was obtained. Again, this strain was transformed with the *pyr4* gene of *N. crassa* to obtain an autotrophic strain (SBV6) for virulence analysis (see below). With SBV2 as recipient strain, only one *nps6-*deletion strain was isolated (SBV4) (Table 4). *nps6* deletion was shown as described above (data not shown). The *nps6* knock-out strain grew indistinguishable from wild type but was not able to grow in the presence of the iron-chelating BPS (bathophenanthrolindisulfonic acid). BPS also inhibits the alternative reductive iron assimilation (RIA) which explains the growth inhibition of the deletion strain under these conditions^12^. In contrast, the Δ*nps2* strain was able to grow, which suggests that the NPS6 enzyme produces the only secreted siderophore (Fig. 1C). Previously, an effect on melanin biosynthesis was reported for the *nps6*-deletion strain^19^. This was not the case in our experiments although many different media were tested. The effect was explained by the fact that some enzymes involved in melanin biosynthesis may require higher amounts of iron^24^. The enzymes in our isolate could be less sensitive than the enzymes of the isolate from citrus^19^.

**Figure 2:**
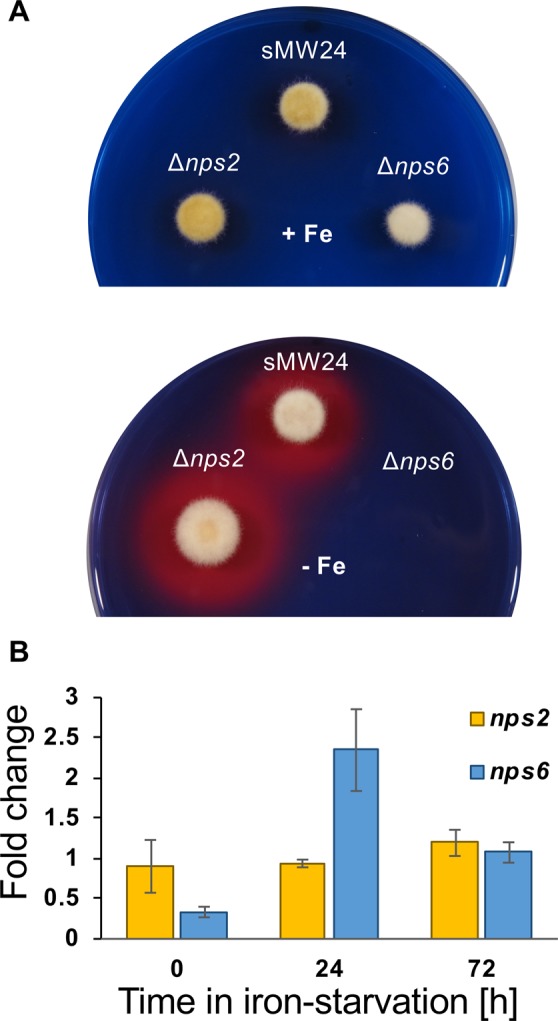
Detection of secreted siderophores in the CAS-assay. (**A**) Growth of the strains indicated on CAS agar plates with and without additional iron. (**B**) Determination of RNA transcript levels by qPCR. Strains were pre-grown for 72 h in mCDB medium containing iron. Afterwards, the mycelium was washed and transferred to fresh medium containing either BPS or not. RNA was extracted from wild type right after the transfer to the starvation medium (time 0) and after 24 and 72 h of iron starvation. The expression was normalized to the housekeeping gene for histone 2B. The expression levels under iron starvation were related to the levels without starvation (fold change). A slight change in expression after the medium change explains that the fold change at time zero is not one. The data represent the mean of three biological replicates. The standard deviation is indicated.

To further characterize the mutant strains, they were grown on CAS agar in the presence or absence of iron (the chelator BPS was added to deplete the medium for iron) (Fig. 2A). The agar contains chrome azurol S, which forms a blue complex with Fe^3+^ and hexadecyltrimethylammonium bromide. If a siderophore removes the iron from the complex, the color changes to orange^25^. The color change on iron-lacking medium was observed in the wild-type strain SMW24 and the *nps2-*deletion strain, suggesting secretion of a siderophore under iron limiting conditions. The *nps6-*deletion strain did not grow in the absence of iron. This was surprising because *A. alternata* should have the high affinity RIA system for iron acquisition. However, BPS also inhibits RIA^12^. Interestingly, after four days of growth, the *nps2-*deletion strain produced a thin orange halo around the colony, indicating siderophore secretion even in the presence of iron (not shown). This was not observed in wild type. Taken together the results indicate that Nps6 produces the extracellular siderophore, whereas Nps2 is required for intracellular siderophore biosynthesis necessary for transport and storage. Apparently, iron storage is required for intracellular siderophore-mediated iron handling and regulation of Nps6 expression regulation. Indeed, whereas the transcript levels of *nps2* were unaffected by iron starvation, *nps6* was significantly upregulated after 24 h (Fig. 2B).

**Figure 3:**
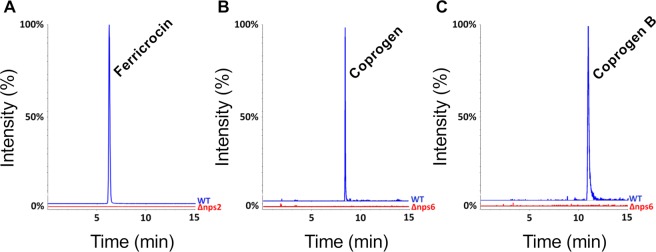
Targeted mass spectrometry (MS) analyses of extracts from wild type, the *nps2* and the *nps6* deletion strain. (**A**) Targeted MS analysis for ferricrocin in cellular extracts of wild type and the *nps2-*deletion mutant. (**B,C**) Targeted MS analysis for coprogen and coprogen B isolated from the medium of wild type and the *nps6-*deletion strain. Each analysis shows the extracted ion track of the corresponding compounds recorded by mass spectrometry. The signal intensity of wild type was set to 100%.

In order to identify which siderophores are produced by NPS2 and NPS6 we compared wild-type and knock-out strain extracts by HPLC-MS analysis. The comparison of cellular extracts of the wild type and the *nps2*-deletion strain showed a peak with the retention time of 6.224 minutes in wild type but not in the mutant strain (Fig. 3A). The MS analysis revealed a mass peak at m/z 771.249 indicative for ferricrocin. Coprogen and coprogen B were unaffected (data not shown). Using the same strategy, we identified coprogen (m/z 822.310) and coprogen B (m/z 780.299) as extracellular siderophores produced by the NRPS Nps6 (Fig. 3B,C). In intracellular extracts, ferricrocine was still detectable in the *nps6-*deletion strain (data not shown).

### Nps2 is involved in spore formation

**Figure 4:**
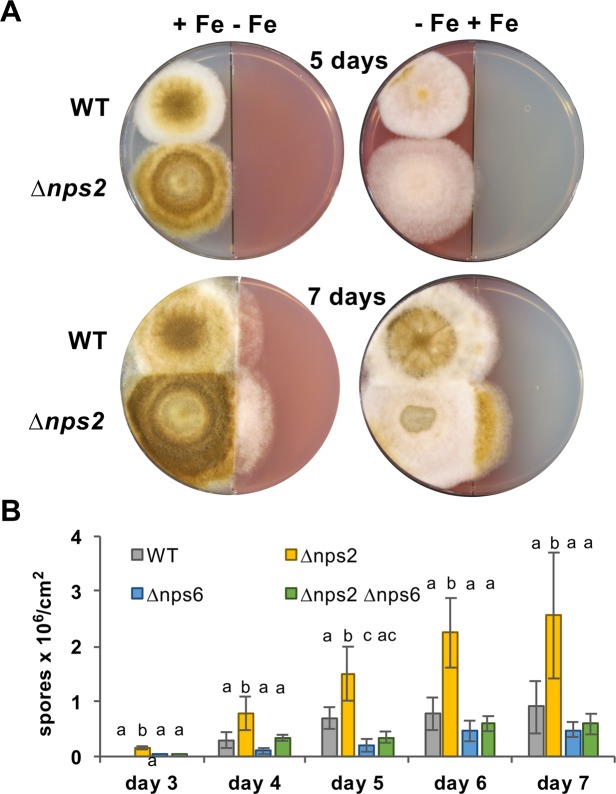
Deletion of *nps2* causes early and increased sporulation. (**A**) WT (SMW24) and the *nps2-*deletion strain (SBV2) were inoculated on split agar plates (plus uracil and uridine) with or without iron as indicated and incubated for 5 or 7 days. (**B**) Quantification of the number of spores on agar plates (plus uracil and uridine) produced after 3 to 7 days. The bars show the mean of three biological replicates. The standard deviation is indicated. A one-way ANOVA with *post hoc* Tukey HSD Test was used to validate significant differences between strains at different time points. Different letters indicate significant differences in spore number/cm^2^ (*p* < 0.05).

Already at the colony level, *nps2-*deletion strains appeared darker than the wild type (Fig. 1C). This was also very evident when wild type and the *nps2*-deletion strain were grown on split agar plates, where either one half was filled with medium containing iron and the other one contained 250 µM of the chelating agent BPS (Fig. 4A). When the strains crossed the border to the iron-containing medium, the *nps2-*deletion strain instantly started sporulation. The number of spores was then quantified after different incubation times on standard agar plates (Fig. 4B). Indeed, the *nps2-*deletion strain produced significantly more spores and started sporulation earlier than the wild type. Intracellular siderophores are used by fungi to store and distribute endogenous iron^3,11^. Intracellular siderophores are also important for the protection of the cells from free iron, which may cause oxidative stress^9^. Ferricrocin fulfils this function in *Aspergillus ochreatus*, *Aspergillus nidulans* and *Neurospora crassa*^3^. This may explain the increased number of spores in the absence of ferricrocine, because reactive oxygen species are important to trigger developmental decisions^26,27^. In comparison, the number of spores appeared slightly lower in the *nps6*-deletion strain as compared to wild type, although the differences were not significant. In *A. alternata* tangerine pathotype, spore formation was reduced to ca. 50% in the *nps6*-deletion strain^19^. This may be related to the fact that also melanin production appeared to be affected in this strain as well. The results suggest that developmental decisions may be very sensitive to small intracellular changes of different metabolites in different species.

### The intracellular iron concentration is important for pathogenicity

**Figure 5:**
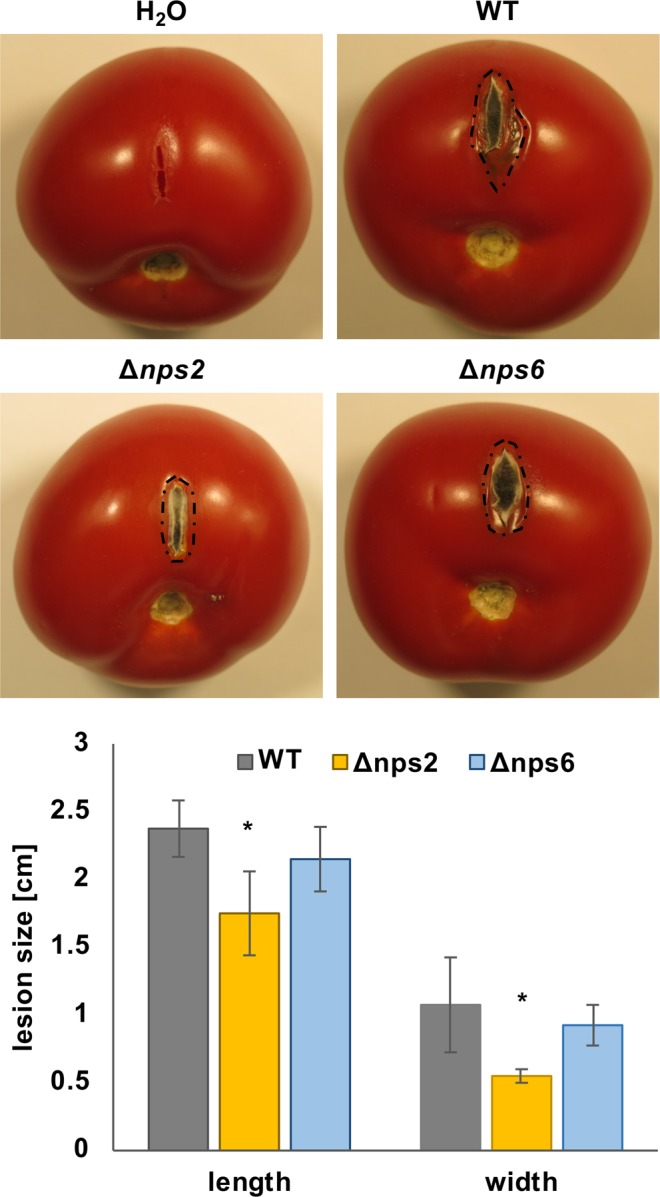
Deletion of *nps2* reduces virulence of *A. alternata*. A 1 cm wide cut was introduced into tomatoes of the same size. 10.000 spores were added to the cut of the WT (ATCC (SBV7), *nps2-*deletion (SBV5) and the *nps6-*deletion strain (SBV6). The tomatoes were incubated for one week at 20 °C. The lesions were measured in length and width. The columns represent the mean of four biological replicates. Two-tailed Student’s t-test was used for pairwise comparisons of lesion sizes. Significance is indicated by an asterisk (^∗^p < 0.05).

There are many examples that siderophores are important during animal and plant infections^1,28,29^. It was shown that deletion of the *nps6* gene in *Cochliobolus heterostrophus, Cochliobolus miyabeanus, Fusarium graminearum*, and *A. brassicicola* on different host plants showed reduced virulence^30^. We tested the virulence of our strains on tomato. The recomplementation of the strains with the pyr-4 gene from *N. crassa* had no effect on virulence (suppl. Fig. S1). After one week the resulting lesions were inspected (Fig. 5). Whereas *nps6* deletion did not affect virulence much, *nps2* deletion caused a reduction of virulence. This result is in agreement with studied in *Metarhizium robertsii*^29^. The fact that the *nps6-*deletion strain grew as well as wild type indicates that external iron is probably not a limiting factor in the case of tomatoes as host plant. In addition, that observation suggests that iron-uptake by the alternative RIA pathway is sufficient for virulence on tomatoes. In a previous study using *A. alternata* strain EV-MIL31, an isolate of diseased leaves of a citrus plant, *nps6* deletion caused a severe reduction of virulence on citrus leaves^19^. The apparent discrepancy to our results may be explained if the citrus isolate is particularly virulent on citrus leaves and iron uptake through the RIA system is not sufficient. However, it can also well be that the iron availability on leaves is very different from the availability in tomatoes, given that in our experiment the fungus did not grow on the tomato surface and did not have to penetrate the cuticle.

In contrast to the slight effect of *nps6* deletion in our experimental set up with tomatoes as host fruits, deletion of *nps2* had a larger effect on virulence. If iron is taken up in the *nps2-*deletion strain, either through RIA or through coprogen, the intracellular concentration of free iron should be tightly controlled through the intracellular siderophore ferricrocine. This is obviously not the case in the *nps2*-deletion strain and probably causes the reduction in virulence. Taken together the results show that iron availability and iron metabolism are intimately linked to fungal metabolism, development and virulence.

## Supplementary information

Suppl. Fig. S1.
