# Supplementary material for: Alternaria alternata uses two siderophore systems for iron acquisition
Source: Sci Rep. 2020 Feb 27;10:3587. doi: 10.1038/s41598-020-60468-7 (PMC7046739; doi:10.1038/s41598-020-60468-7)
Supplement: Supplementary file 1 — Suppl. Fig. S1. [file 41598_2020_60468_MOESM1_ESM.pdf]

Suppl. Material

Alternaria alternata uses two siderophore systems for iron acquisition

Benjamin Voß[1], Frank Kirschhöfer[2], Gerald Brenner-Weiß[2] and Reinhard Fischer[1]*

running head: siderophores in Alternaria

Address: [1] Karlsruhe Institute of Technology (KIT) - South Campus
Institute for Applied Biosciences
Dept. of Microbiology
Fritz-Haber-Weg 4
D-76131 Karlsruhe
Phone: +49-721-608-44630
Fax: +49-721-608-44509
E-mail: reinhard.fischer@KIT.edu
homepage: http://www.iab.kit.de

[2] Karlsruhe Institute of Technology (KIT) - North Campus
Institute of Functional Interfaces
Bioengineering and Biosystems
Hermann-von-Helmholtz-Platz 1
D-76344 Eggenstein Leopoldshafen

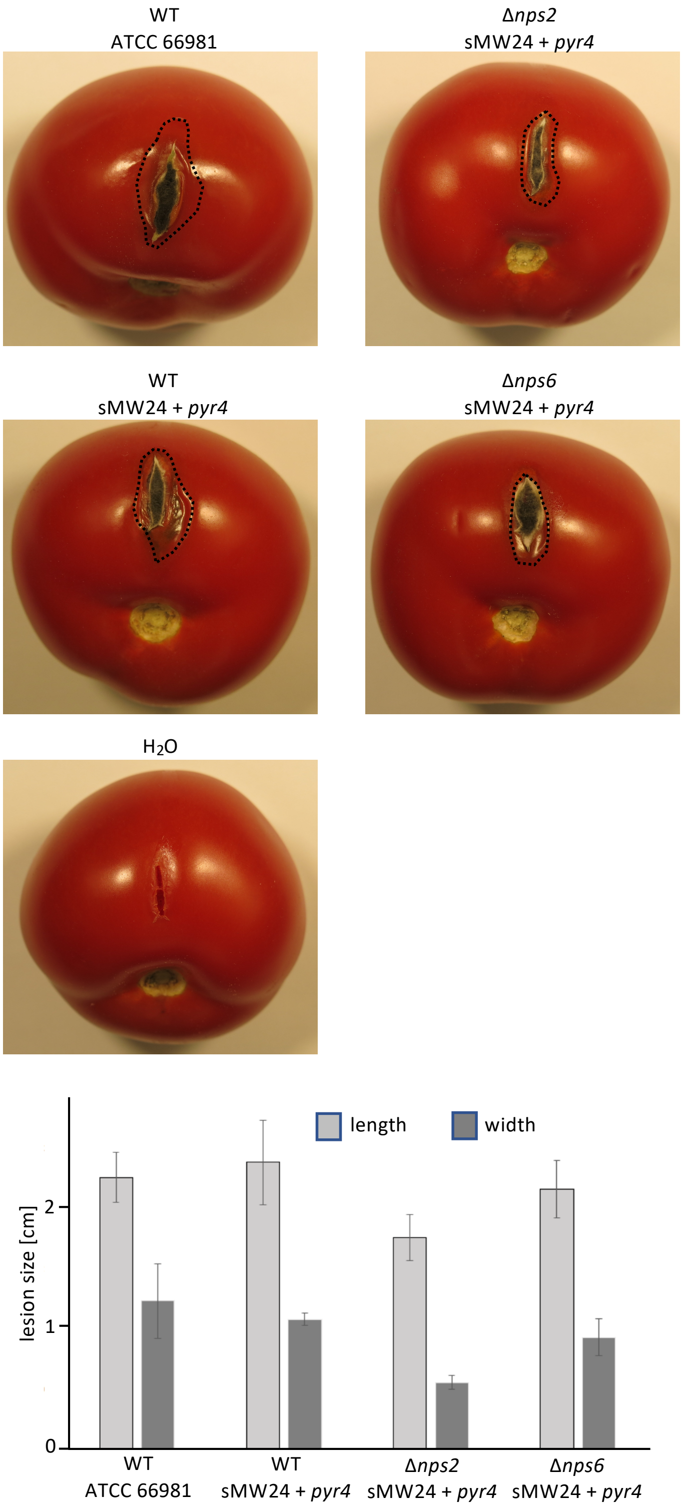

Suppl. Fig. S1: Virulence of *A. alternata* recomplemented strains. A 1 cm wide cut was introduced into tomatoes of the same size. 10.000 spores were added to the cut of the WT (ATCC66981) or WT (SMW24 plus *pyr4,* SBV7), *nps2*-deletion (SBV5) and the *nps6*-deletion strain (SBV6). The tomatoes were incubated for one week at 20 °C. The lesions were measured in length and width. The columns represent the mean of four biological replicates. Two-tailed Student's t-test was used for pairwise comparisons of lesion sizes. Significance is indicated by an asterisk ($*p < 0.05$).
